# Cortico-Cerebellar Hyper-Connections and Reduced Purkinje Cells Behind Abnormal Eyeblink Conditioning in a Computational Model of Autism Spectrum Disorder

**DOI:** 10.3389/fnsys.2021.666649

**Published:** 2021-12-17

**Authors:** Emiliano Trimarco, Pierandrea Mirino, Daniele Caligiore

**Affiliations:** ^1^Computational and Translational Neuroscience Laboratory, Institute of Cognitive Sciences and Technologies, National Research Council, Rome, Italy; ^2^Laboratory of Neuropsychology of Visuo-Spatial and Navigational Disorders, Department of Psychology, "Sapienza" University, Rome, Italy; ^3^AI2Life s.r.l., Innovative Start-Up, ISTC-CNR Spin-Off, Rome, Italy

**Keywords:** autism, associative learning, hyper-connectivity, system-level neuroscience, spiking neuron models, cerebellar-cortical circuit, sensory-motor cortex, prefrontal cortex

## Abstract

Empirical evidence suggests that children with autism spectrum disorder (ASD) show abnormal behavior during delay eyeblink conditioning. They show a higher conditioned response learning rate and earlier peak latency of the conditioned response signal. The neuronal mechanisms underlying this autistic behavioral phenotype are still unclear. Here, we use a physiologically constrained spiking neuron model of the cerebellar-cortical system to investigate which features are critical to explaining atypical learning in ASD. Significantly, the computer simulations run with the model suggest that the higher conditioned responses learning rate mainly depends on the reduced number of Purkinje cells. In contrast, the earlier peak latency mainly depends on the hyper-connections of the cerebellum with sensory and motor cortex. Notably, the model has been validated by reproducing the behavioral data collected from studies with real children. Overall, this article is a starting point to understanding the link between the behavioral and neurobiological basis in ASD learning. At the end of the paper, we discuss how this knowledge could be critical for devising new treatments.

## 1. Introduction

Autism spectrum disorder (ASD) is a neurobiological disorder characterized by difficulties in social communication and restricted behavioral patterns, often including stereotyped or repetitive motor movements, inflexible adherence to routines, and ritualized action practices (Lai et al., 2014; Romanczyk et al., 2016). Further, there may be hyper- or hypo-reactivity to sensory input (Dakin and Frith, 2005; Robertson and Baron-Cohen, 2017) and unusual learning trajectories (Shah and Frith, 1993; White et al., 2009; Baron-Cohen and Lombardo, 2017). In this regard, several works have demonstrated that ASD children show abnormal response on delay eyeblink conditioning (DEBC) (Sears et al., 1994; Oristaglio et al., 2013; Welsh and Oristaglio, 2016). DEBC is a learning paradigm consisting of an association between a conditioned stimulus (CS), typically a tone, and an overlap unconditioned stimulus (US) eliciting eyelid closure, such as an air puff to the cornea. After repeated CS-US pair presentations, conditioned eyelid closure (conditioned response, CR) occurs as a response to CS. Full eyelid closure for the CR typically occurs close to the US onset time (Thompson and Steinmetz, 2009). During DEBC involving ASD children, the CR learning rate is higher in the ASD group than the typical development group (Sears et al., 1994). Additionally, the peak latency, defined as the time between CS onset and the CR signal maximum, occurs significantly earlier for the ASD group (Oristaglio et al., 2013; Welsh and Oristaglio, 2016). The neural mechanisms underlying this atypical learning behavior are not fully clear. This article uses an improved version of the physiologically constrained spiking neuron model of the cerebellar-cortical circuits recently proposed by Caligiore and Mirino (2020) to address this issue. The cerebellum is a fundamental processing unit for various cognitive and motor tasks (Ivry and Baldo, 1992). Several studies have demonstrated the importance of the cerebellum for the acquisition and extinction of CRs in DEBC sessions (see section 2.2.1). The learning capabilities of the cerebellum are related to plasticity mechanisms that change the synaptic weights of connections between different groups of cells (Mar, 1969; Albus, 1971; Ito, 1997). Notably, this work wants to underline the crucial role of cerebellar function from a more complex, systems-level perspective that fully acknowledges its close interplay with different brain areas (Caligiore et al., 2017; Lindeman et al., 2021). In particular, the model aims to demonstrate how two anatomic-physiological features of the autistic brain are critical to explaining the abnormal ASD learning path during DEBC. Firstly, the model reproduces the fewer number of Purkinje cells, often characterizing the autistic brain (White et al., 2009; Skefos et al., 2014; Hampson and Blatt, 2015). Secondly, it reproduces the effects of the cortico-cerebellar hyper-connectivity (Khan et al., 2015; Oldehinkel et al., 2019) also typically present in the autistic brain. The computer simulations run with the model show that the first neural feature is critical to explain the behavioral result on a higher CR learning rate showed by real ASD children (Sears et al., 1994). The second feature is instead critical to explain the results on the earlier peak latency (Oristaglio et al., 2013; Welsh and Oristaglio, 2016). These results represent a first step for understanding the relationship between the behavioral and neurobiological basis of learning in ASD. Notably, this knowledge could be critical for devising new treatments, as discussed at the end of the paper.

## 2. Model

### 2.1. Simulation Tools

The model was developed using the *PyNEST* (Eppler et al., 2009) Python programming language interface of the Neuron Simulation Tool *NEST* (Gewaltig and Diesmann, 2007). In particular, each neuron of the model was modeled through the *iaf*_*psc*_*exp* *NEST* function, reproducing the features of a leaky integrate and fire unit with exponential shaped postsynaptic currents (Tsodyks et al., 2000). The neuron dynamics are numerically integrated based on a computation time step of *t* = 10m. All arriving and transmitted spikes are limited to happen in the resulting time grid steps. Overall, the simulation takes 2,500ms.

Most of the model parameters assume the default values of the *NEST* neuron model *iaf*_*psc*_*exp*, reflecting the values of the related physiological parameters derived from studies with animals or humans. Table 1 summarizes the parameters related to the connections between neurons and those critical to simulate the difference between ASD and control groups. The code of the model is accessible from this link https://github.com/ctnlab/cerebellum_autism_DEBC_model.

**Table 1:** Values of connection weights (*w*), external current (*I*_*e*_) and connections delay parameter (*d*).

** Connection weights**	**External currents**	**Delay parameters (Control/ASD)**
$w_{CS\overset{}{\underset{}{\to }}GR}=500$	*I*_*e*_*GR*__ = 370	$d_{CS\overset{}{\underset{}{\to }}GR}=100/50$
$w_{CS\overset{}{\underset{}{\to }}DN}=500$	*I*_*e*_*PC*__ = 380	$d_{CS\overset{}{\underset{}{\to }}DN}=100/50$
$w_{US\overset{}{\underset{}{\to }}IO}=100$	*I*_*e*_*IO*__ = 370	$d_{US\overset{}{\underset{}{\to }}IO}=100/50$
$w_{IO\overset{}{\underset{}{\to }}PC}=-500$	*I*_*e*_*DN*__ = 370	$d_{DN\overset{}{\underset{}{\to }}M1}=100/50$
$w_{IO\overset{}{\underset{}{\to }}DN}=60$	*I*_*e*_*M*1__ = [300, 365]	
$w_{PC\overset{}{\underset{}{\to }}DN}=-7$	*I*_*e*_*mPFC*__ = [300, 365]	
$w_{DNr\overset{}{\underset{}{\to }}M1}=100$		
$w_{DNp\overset{}{\underset{}{\to }}mPFC}=50$		
$w_{Noise\overset{}{\underset{}{\to }}DN}=\left[0.1,0.5\right]$		
$w_{GRr\overset{}{\underset{}{\to }}PCr}=5$		
$w_{GRp\overset{}{\underset{}{\to }}PCp}=20$		
$w_{mPFC\overset{}{\underset{}{\to }}M1}=0.1$		

*The $w_{Noise\overset{}{\underset{}{\to }}DN}$, I_e_M1__ and I_e_mPFC__ values were randomly chosen in the given range according to a uniform distribution. Thus, each simulated subject has different values for these parameters. The $w_{GR\overset{}{\underset{}{\to }}PC}$ and $w_{mPFC\overset{}{\underset{}{\to }}M1}$ values are those initial since GR-PC and mPFC-M1 connections are plastic*.

### 2.2. Model Architecture and Functioning

**Figure 1:**
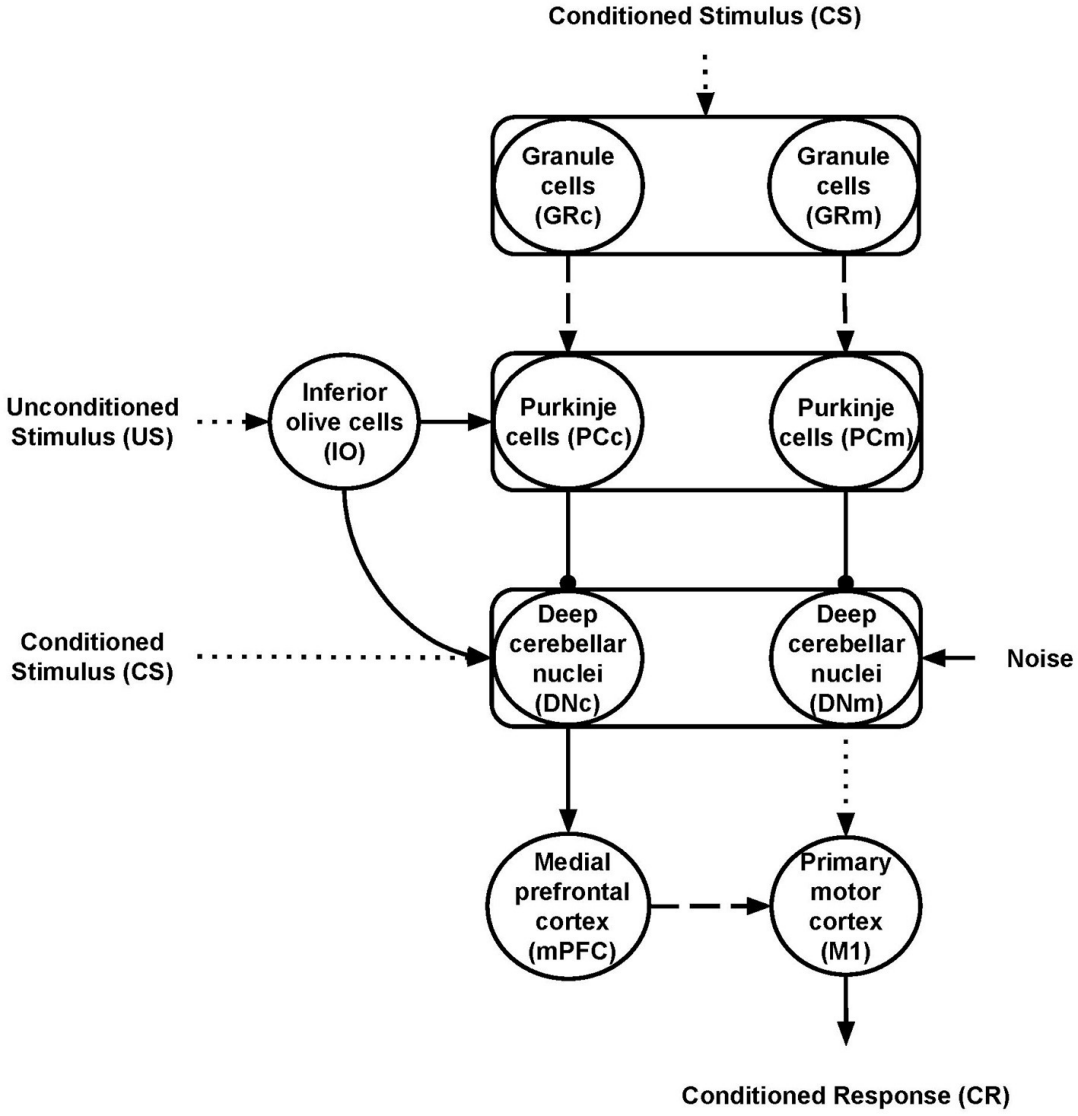
Model architecture. The rectangles indicate the cerebellar regions; the circles represent the cerebellar, inferior olive, and cortical neural populations. The connections linking different areas can be plastic (dashed lines) or fixed (solid lines) or fixed and hyper-connected in ASD model (dotted lines); excitatory (arrows) or inhibitory (lines ending with a dot). The subscripts “m” and “c” indicate the motor and cognitive pathways, respectively.

Nine neural populations of spiking neurons linked through excitatory and inhibitory connections formed the model system-level architecture (Figure 1). Of these, two represent the primary motor cortex (M1) and the medial prefrontal cortex (mPFC). The remaining seven neural populations reproduce the functioning of different parts of the cerebellum. The architecture mainly focuses on the cerebellar anatomical and physiological features while, for simplicity, it does not reproduce the thalamocortical dynamics. Two critical anatomic-physiological components characterize the model architecture: (i) a system-level organisation through parallel cerebellar-cortical circuits (see section 2.2.1); (ii) granule cells subpopulations with different time-sensitivity (see section 2.2.2). Below, we discuss in detail these two features.

#### 2.2.1. Parallel Cerebellar-Cortical Circuits

The cerebellar model builds on well-established spiking neuron architectures (Antonietti et al., 2018; Geminiani et al., 2018). In particular, 1536 Granule cells (GR), 48 Inferior olive cells (IO), 48 Purkinje cells (PC), and 24 Deep cerebellar nuclei (DN) made it. The input signals go to GR and DN (CS) and IO (US) through connection weights, respectively, simulating the signal preprocessing action of mossy and climbing fibers. In this way, the spreading of the activation through the cerebellar regions is only possible if there is some input (CS or US). Otherwise, all the cerebellar regions are silent and, in turn, mPFC and M1 are quiet too. The number of units within each region makes the simulations computationally feasible while resembling the biological ratios (DAngelo et al., 2016). Two parallel cerebellar-cortical circuits anatomically compose the model (Figure 1), each containing half of the total number of neurons: the motor pathway (GRm-PCm-DNm-M1); the cognitive pathway, including mPFC (GRc-PCc-DNc-mPFC-M1).

These two pathways process the signal with a different time-sensitivity (see section 2.2.2 below). Moreover, the cognitive pathway influences the system motor behavior through the connections linking mPFC to M1. This organization agrees with data suggesting that the cerebellum is connected with various parts of the frontoparietal cerebral network through a set of parallel circuits, channels (Middleton and Strick, 2000; Dum and Strick, 2003), managing different cortical contents including, for example, actions or memory patterns (Strick et al., 2009; Caligiore et al., 2013, 2017). In particular, Bernard et al. (2014) firstly report a motor network involving the dorsal dentate, anterior regions of the cerebellum, and the precentral gyrus in the motor cortex and a cognitive network involving the ventral dentate, Crus I, and prefrontal cortex. The motor pathway is essentially involved in DEBC, whereas the cognitive route could have a modulatory role (McCormick and Thompson, 1984; Hardiman and Yeo, 1992; Ernst et al., 2016). Moreover, several data support the influence of the prefrontal region over primary motor areas (Miyachi et al., 2005; Narayanan and Laubach, 2006; Nardone et al., 2019). Some works indicate that M1 is weakly involved in learning during DEBC (Ivkovich and Thompson, 1997), mainly supporting the motor role of the red nucleus (RD) (Pacheco-Caldern et al., 2012). Other studies show precisely the opposite, providing ample evidence for the fundamental role of M1 in modulating CR (Aou et al., 1992; Birt et al., 2003; Ammann et al., 2016) and the auxiliary function of RD (Chapman et al., 1988; Anderson and Keifer, 1997). The RN is quite rudimentary in humans, likely due to the development of the corticospinal tract and the pyramidal system (Ulfig and Chan, 2001; Hicks et al., 2012). The model proposed here intends not to establish which of the two hypotheses is correct but rather to reproduce the core dynamics present in the ASD cerebellum. Notably, the model simulated a central mechanism that explains CR acquisition in DEBC operating within cerebellar circuits before reaching the brain regions that implement movement. Therefore, for simplicity, the model presents only the M1 neural population as the cortical target region of the motor cortico-cerebellar pathway.

#### 2.2.2. Granule Cells Subpopulations With Different Time-Sensitivity

**Figure 2:**
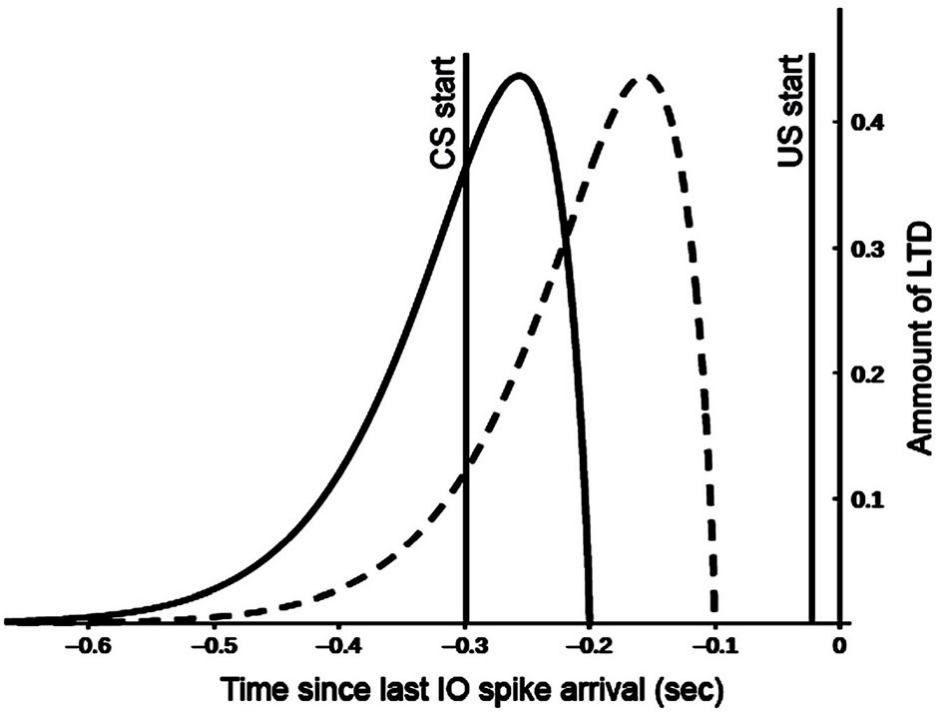
Kernel functions used for GR and PC synaptic long term depression (LTD). Both functions are convolved with the spike train of the afferent parallel fibers (all spikes emitted for *t* < 0 *sec*). This provides a measure of past parallel fibers activity setting the synapse eligibility to depression when the inferior olive (IO) neuron afferent to the PC emits a spike (*t* = −0.02 *sec*). Motor and cognitive kernels are respectively indicated with dashed and solid lines.

The model reproduces one of the most remarkable cerebellum properties: its control in motor operations timing (Mauk and Buonomano, 2004). For this purpose, the model simulates the observed cerebellar granular neurons time-sensitivity according to which different cells are active to varying moments during conditioned stimuli (Medina et al., 2000). The interplay between mossy fibers, granule, and Golgi cells supports this process. According to the time-window matching hypothesis (D'Angelo and De Zeeuw, 2009), the mossy fibers inputs to the granular layer are transformed into well-timed spike bursts by intrinsic granule cell processing. The feedforward Golgi cells inhibition sets a limit to the duration of such a spike. These activities are spread over particular fields in the granular layer to generate ongoing time-windows to control interacting motor domains properly. The different time-sensitivity of granule cells has vast implications for associative learning processes operating within the olivo-cerebellar-cortical system. Indeed, the synaptic plasticity might favor the activation of specific granule cell groups concerning particular time windows. The model uses two temporal kernel functions (Figure 2) to capture the effects of granule cells time sensitivity on long-term depression (LTD) processes operating within the parallel fibers.

These functions correlate the past activity of a single granule cell with each spike from the inferior olive (US) in different ways to construct predictive dynamic responses during associative learning. The IO neurons afferent to the PC emit a spike with *t* = −0.02 *s* because the US stimulus has a duration of 20 *ms* and finishes with the CS stimulus at *t* = 0, to comply with the DEBC paradigm. The "motor" kernel (Figure 2 dashed line) mainly influences the activity of the GRm-PCm-DNm-M1 path and supports high CS-US correlation when the stimulus duration is small (function peak at 150 *ms*). This kernel function starts to produce an effect on the input signal 100 *ms* before IO-spike arrival, in agreement with the physiological delay suggested by the biology (Kettner et al., 1997; Ros et al., 2006). By contrast, the “cognitive” kernel (Figure 2 solid line) mainly modulates the activity of the GRc-PCc-DNc-mPFC-M1 path and allows high CS-US correlation when the stimulus duration is more extended (function peak at 250 *ms*). These features make the model able to process stimuli of different duration and address both trace and delay paradigms (Caligiore and Mirino, 2020). The following equation generates the kernel functions:


(1) $$\begin{array}{r} K\left(t\right)=a\cdot exp\left(-\frac{|\left(t+c\right)\cdot a{|}^{b}}{f}\right)\cdot -sin{\left(\frac{t+c}{e}\right)}^{d} \end{array}$$

where *a* = 15, *b* = 1.8, *d* = 0.75, *f* = 1.3 are parameters used to both normalize the kernel function and to regulate the strength of the associative learning processes, *e* is the Napier number, and *c* is a parameter used to control the function translation along the x-axis (*c* = 0.1 and *c* = 0.2, respectively for the motor and cognitive kernels). The Equation (1) corresponds to a second-order differential system solution and its rationale to model GR time sensitivity can be found in Ros et al. (2006), Carrillo et al. (2008), and Luque et al. (2011). The effects of the different granule cells time-sensitivity propagate over M1 and mPFC, supporting these cortical areas functioning at different time-scale, with M1 processing information faster than mPFC (Kiebel et al., 2008).

#### 2.2.3. Connections

The motor and cognitive pathways have the same cerebellar anatomical organisation. For each pathway, GR units receive CS and are connected to PC neurons through the parallel fibers. The IO neurons process US and project to PC through the climbing fibers (Thompson and Steinmetz, 2009). Both CS and US are spike trains generated with the *NEST* function *spike*_*generator*, setting a spike frequency of 100 spikes per second (*sp*/*s*). PC neurons combine the information coming from both GR and IO. The DN neurons represent the cerebellar output. This area receives CS, excitatory signals from IO and inhibitory connections from PC (Dum and Strick, 2003; DAngelo et al., 2016). The DN neurons belonging to the motor and cognitive pathways project, respectively, to M1 and mPFC (Kelly and Strick, 2003). Finally, mPFC projects to M1 modulating its activity (Miyachi et al., 2005). The average firing rate of M1 neurons represents the CR. Aside from the IO-PC connections, which are “one-to-one,” the connections linking the model areas are “all-to-all.”

All neurons are stimulated by an external current *Ie* simulating the effects of the external signals supplied by other areas not reproduced in the model (Tsodyks et al., 2000). For each model area, we set the values of *Ie* to pre-activate cells avoiding at the same time too spurious activity covering the effects of the main signals CS and US. Also, we used a noise signal (*Noise*) to stimulate DN neurons, simulating the spurious effects on neural activation due to the intrinsic neural noise (Schweighofer et al., 2004). Spike train, generated through a Poisson process having a given frequency rate, represents the *Noise*. This assumption agrees with empirical evidence and models showing that Poisson processes approximated cortical spikes temporal distribution (Poznanski, 2011). The *NEST* function *poisson*_*generator* simulated the Poisson process with the following parameters: mean firing rate (rate = 2500 *sp*/*s*); time origin of the simulation (origin = 1 *ms*); beginning of device application to origin (start = 1 *ms*); termination of device application to origin (stop = 2, 500 *ms*). Within the model nine synaptic connections are static (CS-GR, CS-DN, US-IO, IO-PC, IO-DN, PC- DN, DN-M1, and DN-mPFC) (Figure 1, solid or dot lines) while the other two (GR-PC, mPFC-M1) are plastic (Figure 1, dashed lines). Table 1 summarizes the *Ie* values and the connections parameters used in the model. The Table 1 also shows the connections *delay* parameters we used to reproduce the effects of different connectivity between ASD and the control group (see section 2.2.5 for more details).

#### 2.2.4. Plasticity Mechanisms

The plasticity rules described below drive the weights change of the plastic connections during the training sessions, increasing the weights by long term potentiation (LTP), or decreasing them by long term depression (LTD). The LTD implemented at the GR-PC synapses is an associative weight decrease triggered by spikes from IO (Ito, 2001). The LTD algorithm uses the temporal kernels shown in the Figure 2, which correlate each spike from IO (US) with the past activity of GR (CS) (Caligiore et al., 2019a; Caligiore and Mirino, 2020). The spike train supplied to the GR-PC afferent connection (all CS spikes emitted for *t* < 0 *s* in the Figure 2) is separately convolved with both motor and cognitive kernels. In this way, it is possible to have a measure of past parallel fibers activity that is used to set the synapse eligibility to depression when the IO neurons afferent to the PC emit a spike (from *t* = −0.02 *s* to *t* = 0.0 *s* in the Figure 2). This rule maximizes learning (LTD) at synaptic sites in which the input parallel fibers delayed activity positively correlates with the IO signal. Hence, the kernel functions showed in the Figure 2 help the cerebellum to acquire the capacity to produce a predictive output. This feature is critical in associative sensory-motor paradigms, such as delay or trace eyeblink conditioning. In this case, indeed, the cerebellum learns to predict the precise timing between two stimuli, CS and US, and produces a CR precisely timed to anticipate the US onset (DAngelo et al., 2016). Non-associative weight increase implements the LTP at the GR-PC synapses (Lev-Ram et al., 2003). The long term plasticities for the GR-PC connections are responsible for CR acquisition (LTD) and extinction (LTP) (Antonietti et al., 2016). Below the equation regulating the GR-PC LTD and LTP plasticity processes:


(2) $$\Delta w_{GR_{i}\to PC_{j}}(t)=\left\{ \begin{array}{l} -\int_{-\infty }^{t_{IO}}K(t-x)\delta_{GR_{i}}(t-x)dx\\ if\;PC_{j}\;is\;active\;and\;t=t_{IO}\\ \alpha \;if\;PC_{j}\;is\;active\;and\;t\neq t_{IO}\\ 0\;otherwise \end{array}\right.$$

where *t*_*IO*_ is the time of the last IO spike arrival; *K* is the integral kernel function that for learning within the motor pathway has its peak at 150 *ms* before *t*_*IO*_, whereas for learning within the cognitive pathway has its peak at 250 *ms* before *t*_*IO*_; δ_*GR*_(*t*) is the Dirac function representing the CS spike train on *GR*_*i*_ cell; α is the LTP learning rate set to 0.05.

Regarding the learning processes modulating the value of the PFC-M1 connection weights, if activation of mPFC is detected 0.04 *s* before the activity of M1, then increases the value of the connection weights between the mPFC-M1 synapses (LTP) (Sjöström et al., 2001; Nevian and Sakmann, 2006). In this way, we assume that the spike in mPFC contributes to generating the spike on M1. Otherwise, there is LTD. Below the equation regulating these learning mechanisms:


(3) ΔwmPFCi→M1j={β if M1j is activeand tmPFCi∈[tM1j−0.04,tM1j]γ if M1j is activeand tmPFCi∈[tM1j−0.04,tM1j]0  otherwise

For each simulated subject, β and γ are randomly chosen according to a uniform distribution, respectively, in the [0.2, 0.5] and in the [−0.015, −0.035] ranges; *t*_*mPFCi*_ and *t*_*M*1*j*_ are the time of the spike occurring, respectively, within the *mPFCi* and *M*1*j* cells.

Before associative learning, the weights of the GR-PC connections have positive values. In this case, a CS produces a great activity within PC layers, which generates a strong inhibition of DN units. During associative learning, the LTD process gradually reduces inhibition from PC to DN (Ishikawa et al., 2014). The consequent DN activity, in turn, contributes to obtain a greater activation of M1 (motor pathway) producing CR, and of mPFC (cognitive pathway). The GR-PC LTD (Equation 2) is responsible for CR acquisition, whereas the mPFC-M1 LTP (Equation 3) makes the influence of mPFC on M1 activity stronger after each training session (see section 3.3 for more details).

#### 2.2.5. Modeling Differences Between ASD Group and Control Group

The ASD group consists of computational models that diverge from the models used to simulate the control group in two features: (i) reduced number of Purkinje cells (Whitney et al., 2009; Skefos et al., 2014; Hampson and Blatt, 2015) and (ii) hyper-connectivity of the cerebellum with sensory and motor cortex (Khan et al., 2015; Oldehinkel et al., 2019). To computationally reproduce (i), we reduced the PC number of both pathways from a population of 48 units to one of 30 units. This reduction rate agrees with literature indicating that autistic brains show 24–50% fewer of Purkinje cells (Fatemi et al., 2002). To simulate (ii), we modulated the signal transmission speed by tuning a *delay* parameter connecting different neural populations. We assumed that the hyper-connected connections have a lower delay in signal transmission. Thus, to reproduce the ASD hyper-connection of the cerebellum with sensory and motor cortex, we reduced the *delay* parameter from 100 to 50 *ms* (see Table 1). The connections involved in the hyper-connectivity of the cerebellum with sensory and motor cortex are CS-GRm, CS-GRc, CS-DNm, CS-DNc, US-IO, and DNm-M1.

### 2.3. Training Protocols

We used DEBC protocols with 10 training sessions. Each training session consists of three trials. Each trial starts just after the previous one ends. Similarly, each training session begins just after the last one ends. Standard training trials consist of 300 *ms* CS with 20 *ms* US final overlapping. The delay protocol allows controlling if the model reproduces behavioral data about the CR learning rate, which is higher in the ASD group than in the typical development group, and the CR peak latency of the ASD group that occurs significantly earlier than those of the control group.

Two groups of 15 simulated children each were trained using the protocol described above. One represents the "control group" formed by healthy children models; the other represents the "autistic group" formed instead by models with a reduced number of Purkinje cells and hyper-connectivity of the cerebellum with sensory and motor cortex (see section 2.2.5).

The model simulates different children using various *NEST* random number generator seeds to produce different noise signal values and different model parameters whose values were randomly drawn from a uniform distribution (see Table 1). The model generates data comparable to those drawn from experiments with real children devised by Sears et al. (1994), Oristaglio et al. (2013), and Welsh and Oristaglio (2016). These data are relevant because they provide the first report of abnormal conditioned response on DEBC in ASD.

## 3. Results

This section shows the data obtained through the simulations run with the model and aiming at: (i) reproducing the main results on a higher CR learning rate and faster timing-response (Peak Latency - PL) obtained with real ASD children involved in DEBC experiments (Sears et al., 1994; Oristaglio et al., 2013; Welsh and Oristaglio, 2016); (ii) understanding the system-level neural mechanisms underlying such results.

### 3.1. Higher CR learning rate on DEBC in ASD

We first tested the ability of the groups to acquire CRs during the DEBC task. For each training session, the *CR Rate* (%) was computed according to the following equation:


(4) $$\begin{array}{r} CR\;Rate\;\left(\%\right)=\frac{<FR_{M1}>\times 100}{FR_{{M1}_{max}}} \end{array}$$

where < *FR*_*M*1_> and *FR*_*M*1_*max*__ are, respectively, the average and the maximum M1 firing rates. These values are calculated in a separated "test phase" at the beginning of each training session, where there is only the CS signal in the system. In the test phase, CR is computed in the [0, 450] *ms* time interval for the control group and in the [0, 400] *ms* time interval for the ASD group. This choice of using two different time intervals was made to accurately capture the firing rate related to the CR and not to other stimuli produced by the noise.

**Figure 3:**
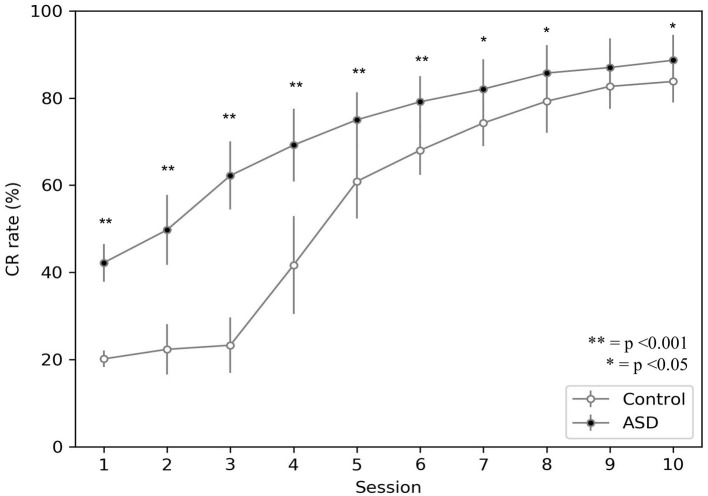
Acquisition of conditioned response during DEBC by simulation. Data obtained with groups of 15 simulated subjects over 10 training sessions. We compared the CR of subjects of the two groups, as the average of each session. The distribution does not respect the assumptions for the use of parametric tests. Applying the Mann-Whitney *U*-test to all sessions, the difference is significant for all sessions except for session 9. Respectively *p* < 0.001 for sessions from 1 to 6; *p* = 0.005 for session 7; *p* = 0.030 for session 8; *p* = 0.067 for session 9; *p* = 0.021 for session 10. Note that since we have two sets of non-parametric sample data, we use the Mann-Whitney *U*-test to test the null hypothesis without correction for multiple comparisons.

Figure 3 shows the behavior acquired by the two groups during DEBC tasks. In particular, it compares the average CR rate of each subject of the control and ASD groups. Like the results obtained through experiments involving real subjects (Sears et al., 1994), even with the model, the percentage of CRs is higher in the ASD group than in the control group.

**Figure 4:**
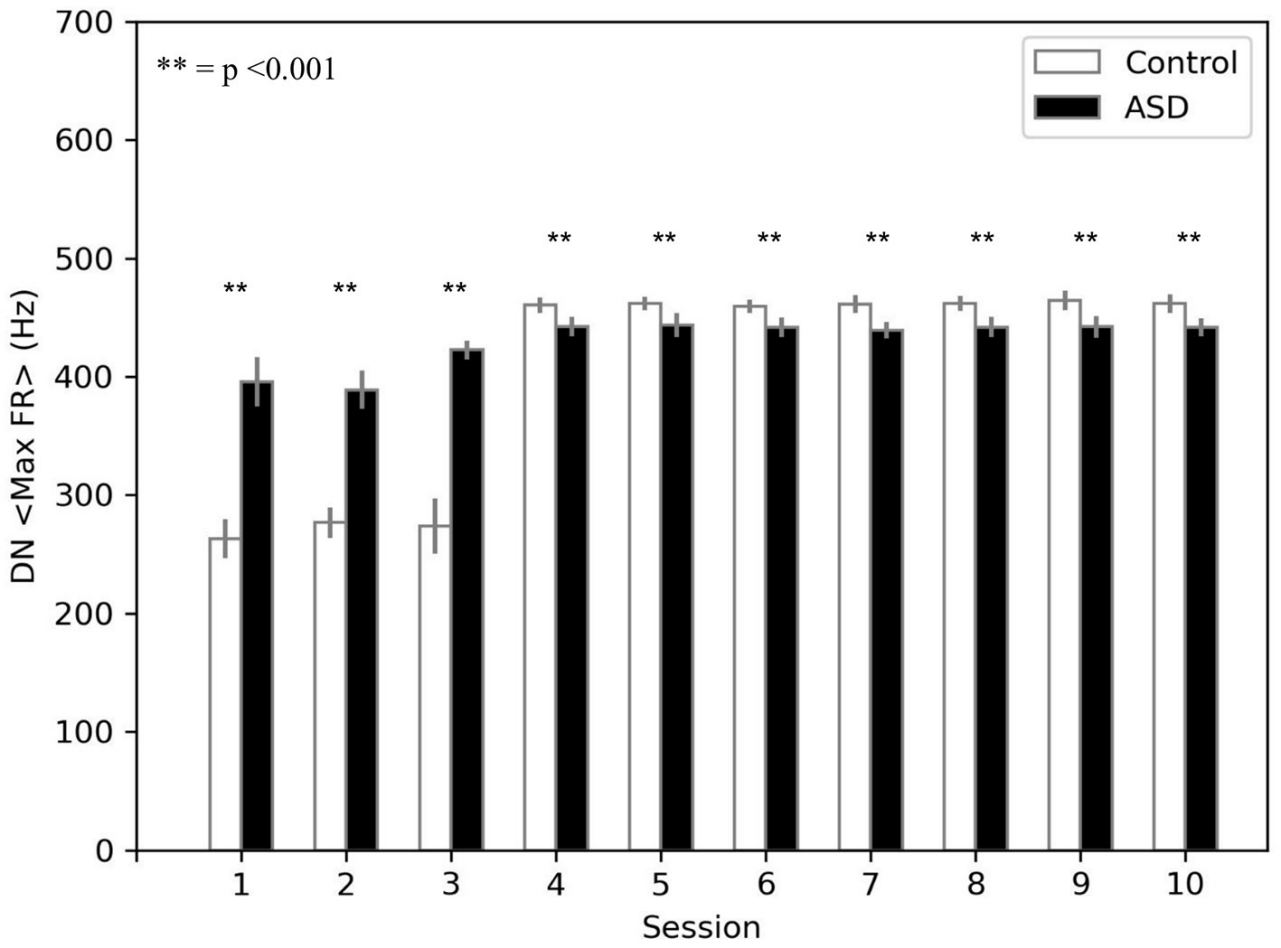
Average max firing rate of dentate nuclei (both DNm and DNc) (*DN* < *MaxFR* >) during DEBC. Data obtained with groups of 15 simulated subjects over 10 training sessions. We compared *DN* < *MaxFR* > of subjects of the two groups, as the average of each session. The distribution does not respect the assumptions for the use of parametric tests. Applying the Mann-Whitney U test to all sessions, the difference is significant for all sessions *p* < 0.001.

The model suggests that the neural mechanism mainly contributing to obtain this behavioral result is the reduced number of PC in ASD. In this respect, Figure 4 suggests that a reduced number of PC leads to reduced DN inhibition, which shows an early higher activation for ASD (fewer learning sessions are sufficient to obtain the DN disinhibition). Consequently, earlier disinhibition of DN causes an earlier activation of M1 and, in essence, an increase in the percentage of CR in fewer sessions in ASD (see Equation 4).The difference of DN activation between the two groups vanishes and even changes direction after PC learning, favoring the control group to recover the CR expression gap. Notably, another critical mechanism in CR expression is the increase in weight between mPFC and M1, which plays a role in the variation in CR expression after PC learning (see section 3.3).

### 3.2. Anticipatory Peak Latency on DEBC in ASD

**Figure 5:**
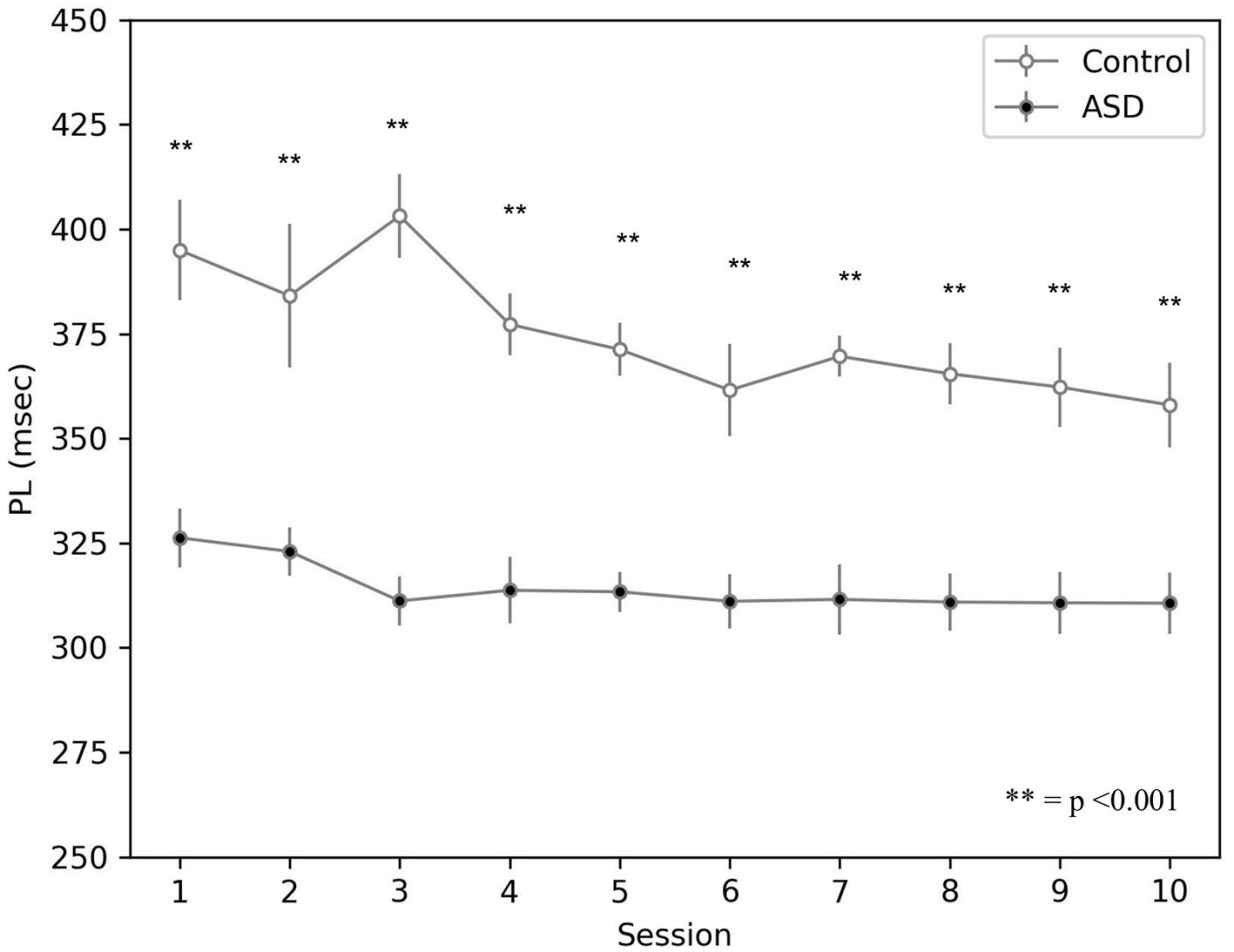
Peak latency response during DEBC by simulation. Data obtained with groups of 15 simulated subjects over 10 training sessions. We compared the PL of subjects of the two groups, as the average of each session. The distribution does not respect the assumptions for the use of parametric tests. Applying the Mann-Whitney *U*-test to all sessions, the difference is significant for all sessions *p* < 0.001.

The simulations run with the model show that the CR peak latency values are lower for the simulated ASD group (Figure 5). We obtained the peak latency (*PL*) by averaging the time when the maximum value of the M1 firing rate occurs (*t*_*F*_*R*__*M*1__) over the time steps (*n*) included in a specific time window, which is [0, 550] *ms* for the control group and [0, 450] *ms* for the ASD group. We use two different time intervals to accurately reflect the timing of the M1 firing rate related to the CR and not to other stimuli generated by the noise. Below the equation used to calculate the peak latency:


(5) $$\begin{array}{r} PL=\frac{\sum_{i=0}^{n}t_{FR_{M1}}}{n} \end{array}$$

The result showed on Figure 5 agrees with data collected with real ASD and control subjects (Sears et al., 1994; Oristaglio et al., 2013; Welsh and Oristaglio, 2016) and indicates that CR signal reaches the peak faster for the simulated ASD group with the same training trial.

**Figure 6:**
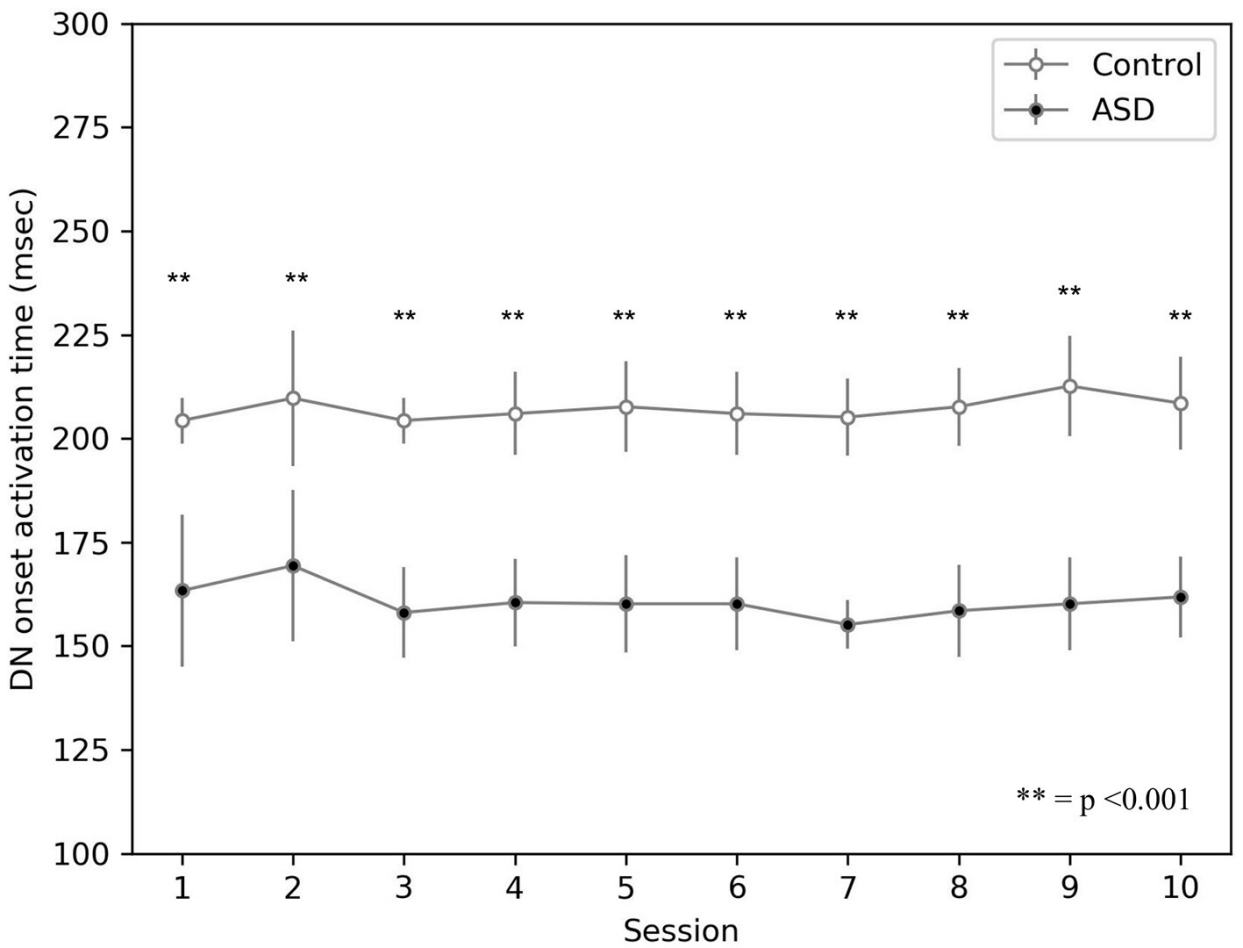
Average timing firing rate of dentate nuclei (both DNm and DNc) during DEBC. Data obtained with groups of 15 simulated subjects over 10 training sessions. We compared the DN timing firing rate of subjects of the two groups, as the average of each session. The distribution does not respect the assumptions for the use of parametric tests. Applying the Mann-Whitney *U*-test to all sessions, the difference is significant for all sessions *p* < 0.001.

The model suggests that the neural mechanism contributing to this behavioral result is the hyper-connectivity between the cerebellum and sensory-motor network in ASD. In this respect, Figure 6 shows that this hyper-connectivity leads to fast DN disinhibition. Consequently, earlier disinhibition of DN causes an earlier activation of M1 and, in essence, lower CR peak latency values in the simulated ASD group.

### 3.3. Brain Mechanisms Underlying ASD Behavior and mPFC Involvement in DEBC

**Figure 7:**
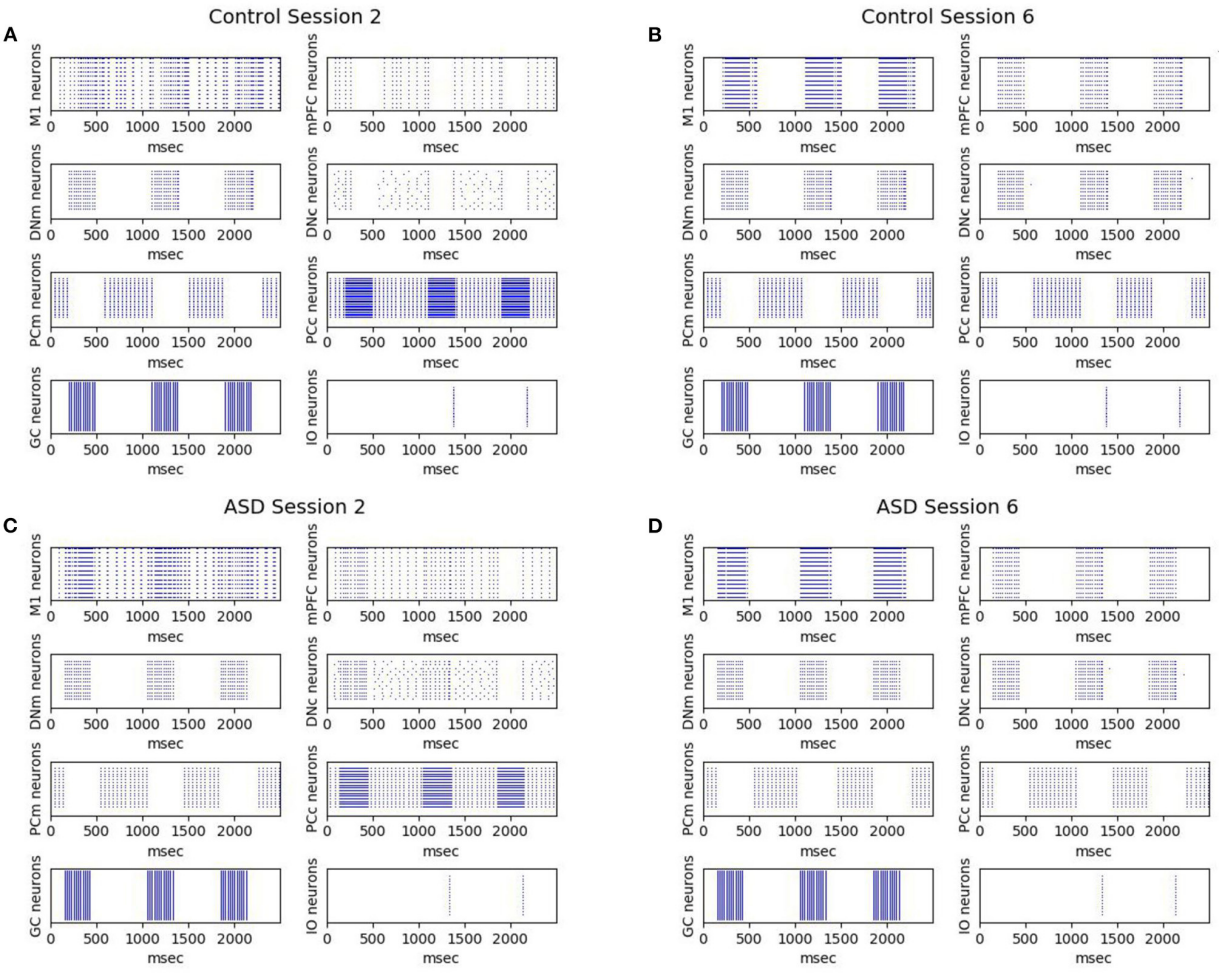
Neurons activation during DEBC simulation. Data obtained during session 2 **(A,C)** and session 6 **(B,D)**, respectively by the control group **(A,B)** and the ASD group **(C,D)**.The comparison between **(A,B)** and **(C,D)** shows robust and rapid activation of M1 in the ASD group rather than in the control group.

Figure 7 shows the effects on the neural activity of the two brain features characterizing the autistic phenotype. First, the lower number of PC in ASD influences the earlier greater activation of DN (see Figure 4) and consequently the earlier greater activation of M1, from which is calculated the CR (see Equation 4). Comparing the activation times of the two groups, we can also see an earlier (and greater) activation in those of the ASD group, particularly in M1, from which is calculated the peak latency (see Equation 5). For these neural dynamics, in the ASD group, the percentage of CRs is higher, and the CR signal reaches the peak faster than the control group. For both ASD and control groups, Figure 7 also shows that after a few sessions (Figures 7A,C), the LTD processes lead to getting a tangible inhibition of only the PCm belonging to the motor pathway. In contrast, the PCc of the cognitive pathway becomes inhibited only with the progression of learning (Figures 7B,D). The M1 activity is initially mainly supported by the motor pathway and then also by the cognitive path. Thus, mPFC (cognitive path) exerts only a modulatory influence on the M1 activity only after a few repetitions and not from the beginning. In this way, the model suggests possible neural dynamics underlying the involvement of PFC in associative learning processes found in empirical experiments (Nardone et al., 2019). The model also suggests that the neural processes supporting the mPFC involvement in DEBC could be influenced by both the greater functional connectivity between DN and mPFC (simulated by the lower DNc-mPFC delay parameter) and the reduced connectivity with M1 (simulated by the higher DNm-M1 delay parameter) (Allen et al., 2005; Habas, 2010; Bostan et al., 2013).

## 4. Discussion

The simulations run with the model show that the autistic brain features reproduced by the model, namely the reduced number of Purkinje cells and the hyper-connectivity of the cerebellum with sensory and motor cortex, are critical to explaining the experimental data about DEBC learning in ASD. In particular, the higher ASD CR learning rate found from real children study (Sears et al., 1994) and replicated by the computational model (Figure 3) could be due to a reduced number of Purkinje cells. The consequence of this loss is more powerful disinhibition of the dentate nucleus (Figures 4, 7), which in turn facilitates the associative learning processes along the motor pathway of the model in ASD. Note how the associative learning processes operating within the cognitive pathway and mainly involving the mPFC-M1 circuits, critically contributes to the gradual improvement of CR acquisition for both ASD and control groups. Therefore, the cognitive pathway becomes more involved with learning, as shown in the Figure 7. Interestingly, this latter result agrees with recent data supporting the involvement of PFC in DEBC (Nardone et al., 2019) and suggests a possible neural mechanism on how PFC could contribute to associative learning processes.

The result about lower peak latency found in experiments with real children (Sears et al., 1994; Oristaglio et al., 2013; Welsh and Oristaglio, 2016) and reproduced by the model (Figure 5) mainly depends on the hyper-connection of the cerebellum with sensory and motor cortex. In the model, the effects of this hyper-connection are reproduced manipulating the connection delay parameter, affecting the signal transmission speed between different neural populations. There is a higher transmission rate in the connections between the areas where CS and US originate and the cerebellum, so the latter receives sensory input earlier in the ASD group than in the control group (Figure 6). Similarly, the hyper-connectivity between the dentate nucleus belonging to the motor pathway and the motor area allows a fast M1 uploading in the ASD group compared to the control group.

Building on these results, new methodologies could be devised to act on these neural processes, for example, to manipulate the degree of hyper-connectivity. In this respect, transcranial magnetic stimulation (Demirtas-Tatlidede et al., 2013) or transcranial direct current stimulation (D'Urso et al., 2015) can be applied as therapeutic modalities in ASD subjects to reduce the effects of hyper-connectivity and to modulate synaptic plasticity. Besides, hyper-connectivity could be manipulated through drug treatments, such as Memantine, NMDA receptor antagonist, that have already tested in ASD to restore the imbalance between excitation and inhibition (Ghaleiha et al., 2013; Uzunova et al., 2014). All of these methodologies could be incorporated into future versions of the model to test their effectiveness.

### 4.1. Related Works

Several theories underlying ASD have been formulated over the years (Fakhoury, 2015), and some of them support our model (Belmonte et al., 2004; Baron-Cohen et al., 2009; Markram and Markram, 2010). Our hypothesis is in line with the numerous studies related to the abnormal cerebellum (Hampson and Blatt, 2015) and its hyper-connectivity with the sensory and motor cortex in ASD (Khan et al., 2015; Oldehinkel et al., 2019).

ASD subjects could show deficits in long-range connectivity with cortical sites, producing, in turn, impairments in cognitive functions coordination (Courchesne, 1997; Fatemi et al., 2002; Verly et al., 2014). Recent genetic (Gharani et al., 2004) and MRI-behavior correlation (Akshoomoff et al., 2004; Kates et al., 2004) studies suggest that cerebellar abnormality may play a more central role in ASD than previously thought. The reduction in Purkinje cell numbers would release the deep cerebellar nuclei from inhibition, producing abnormally strong physical connectivity and potentially abnormally weak computational connectivity along the cerebello-cortical circuit (Belmonte et al., 2004).

Our model agrees with the *Intense* *World* *Theory* (Markram and Markram, 2010), suggesting that hyper-sensitivity could result from a processing difference at various sensory levels. This difference could include the density or sensitivity of sensory receptors, inhibitory and exhibitory neurotransmitter imbalance, or neural processing speed. Besides, Belmonte and colleagues suggested that local range neural overconnectivity in posterior, sensory parts of the cerebral cortex are responsible for the hyper-sensoriality in people with ASD (Belmonte et al., 2004). Studies investigating the sensory profile have revealed sensory abnormalities in over 90% of children with ASD (Kern et al., 2006; Leekam et al., 2007; Tomchek and Dunn, 2007). Furthermore, numerous studies report abnormal perception in ASD in different sensory channels (Bertone et al., 2003; Cascio et al., 2008; Jrvinen-Pasley et al., 2008). In particular, ASD showed hyper-sensitivity to vibrotactile stimulation in the *tactile* modality (Blakemore et al., 2006) and superior pitch processing in the *auditory* modality (Mottron et al., 1999; Bonnel et al., 2003). In addition, recent works support the imbalance of excitation and inhibition in the neocortex in ASD (Hussman, 2001; Casanova et al., 2003; Rubenstein and Merzenich, 2003), with excitation winning over inhibition. In particular, suppressed GABAergic inhibition and increased glutamatergic excitation (Uzunova et al., 2016).

The model proposed here does not reproduce some aspects, such as some neurotransmitter modulatory action (Goris et al., 2020) and the imbalance of excitation and inhibition in the neocortex (Hussman, 2001; Casanova et al., 2003). By contrast, the model successfully captures the evidence on the crucial role of the cerebellum and altered sensoriality in ASD and demonstrates that these features are critical to investigate abnormal EBC behavior in ASD.

## 5. Conclusion

Building on a computational modeling approach, this work proposes that two anatomic-physiological features of the autistic cerebellar-cortical network, the fewer number of Purkinje cells (Whitney et al., 2009; Skefos et al., 2014; Hampson and Blatt, 2015), and the hyper-connectivity between the cerebellum and sensory-motor network (Khan et al., 2015; Oldehinkel et al., 2019), are critical to explaining the neural mechanisms underlying the ASD abnormal behavior in DEBC. In more detail, the simulated subjects behavior is consistent with the experimental observations in real subjects (Sears et al., 1994; Oristaglio et al., 2013; Welsh and Oristaglio, 2016). Moreover, the biological plausibility of model allowed us to formulate hypotheses on the low-level neural mechanisms underlying DEBC and to explore the relationships between ASD brain neuroanatomy and altered behavior.

Notwithstanding these positive features, future works could improve the model in several ways. Among these, the introduction of more complex neuromodulatory mechanisms could provide additional information about the detailed neurobiological processes underlying ASD. In other words, an enhanced version of the model could directly simulate the action of noradrenaline, dopamine and acetylcholine (Lawson et al., 2017), manipulating, for example, the responsiveness of their associated receptors (Caligiore et al., 2019b). We can also investigate the role of the environment in ASD learning. In this respect, behavioral results show that performance in volatile environments is lower in participants with more autistic traits (Goris et al., 2020). Finally, the system-level hypothesis proposed by the model could be tested through new experiments. For example, it could be devised an experiment to compare the behavior of three groups: typical development, low and high functioning ASD children involved in DEBC and trace eyeblink conditioning (TEBC) tasks. In this way, it could be possible to investigate changes in the timing performance of CR acquired during trace and delay eyeblink conditioning in subgroups of ASD children. This investigation could be useful in studying the differences in response timing between ASD subgroups during DEBC and understanding why autistic functioning does not diverge from that of the control group during TEBC (Oristaglio et al., 2013; Welsh and Oristaglio, 2016).

## Data Availability Statement

The datasets presented in this study can be found in online repositories. The names of the repository/repositories and accession number(s) can be found below: https://github.com/ctnlab/cerebellum_autism_DEBC_model.

## Author Contributions

ET, PM, and DC: conceptualization, data curation, investigation, methodology, software, validation, writing–review, and editing. ET and PM: formal analysis and resource. DC: funding acquisition and project administration. PM and DC: supervision. ET and DC: writing–original draft. All authors contributed to the article and approved the submitted version.

## Funding

This research was supported by the ERASMUS + project ARIS (www.aris-project.eu), Grant Agreement 2019-1-BE01-KA202-050425, and by the Advanced School in Artificial Intelligence (www.as-ai.org).

## Conflict of Interest

PM and DC were employed by the company AI2Life s.r.l. The remaining author declares that the research was conducted in the absence of any commercial or financial relationships that could be construed as a potential conflict of interest.

## Publisher's Note

All claims expressed in this article are solely those of the authors and do not necessarily represent those of their affiliated organizations, or those of the publisher, the editors and the reviewers. Any product that may be evaluated in this article, or claim that may be made by its manufacturer, is not guaranteed or endorsed by the publisher.
